# Setting priorities in health care organizations: criteria, processes, and parameters of success

**DOI:** 10.1186/1472-6963-4-25

**Published:** 2004-09-08

**Authors:** Jennifer L Gibson, Douglas K Martin, Peter A Singer

**Affiliations:** 1 University of Toronto Joint Centre for Bioethics, 88 College Street, Toronto, Ontario, M5G 1L4, Canada; 2 Department of Health Policy, Management and Evaluation, University of Toronto, 88 College Street, Toronto, Ontario, M5G 1L4, Canada; 3 Department of Medicine, University of Toronto, 88 College Street, Toronto, Ontario, M5G 1L4, Canada

## Abstract

**Background:**

Hospitals and regional health authorities must set priorities in the face of resource constraints. Decision-makers seek practical ways to set priorities fairly in strategic planning, but find limited guidance from the literature. Very little has been reported from the perspective of Board members and senior managers about what criteria, processes and parameters of success they would use to set priorities fairly.

**Discussion:**

We facilitated workshops for board members and senior leadership at three health care organizations to assist them in developing a strategy for fair priority setting. Workshop participants identified 8 priority setting criteria, 10 key priority setting process elements, and 6 parameters of success that they would use to set priorities in their organizations. Decision-makers in other organizations can draw lessons from these findings to enhance the fairness of their priority setting decision-making.

**Summary:**

Lessons learned in three workshops fill an important gap in the literature about what criteria, processes, and parameters of success Board members and senior managers would use to set priorities fairly.

## Background

Hospitals and regional health authorities in Canada and elsewhere are facing significant resource allocation challenges. Priorities must be set among competing opportunities because demand for health care exceeds available resources. Board members and senior administrators are looking for practical ways to improve how they set priorities under resource constraints. The priority setting literature describes priority setting in various health care contexts [1-9]. It identifies a number of decision-making principles and approaches that could be used to set priorities [10-16]. However, very little has been reported from the perspective of Board members and senior administrators themselves about what decision-making elements (criteria and processes) they would find most useful in setting priorities or how they would evaluate the success of a priority setting exercise.

Fairness is a key ethical goal of priority setting when health care resources are scarce. Experience shows that there is often disagreement on what principles should be used to make fair allocation decisions (i.e., distributive fairness) [8,17]. This means that decision-makers must rely instead on a fair process (i.e., procedural fairness) to establish the legitimacy of priority setting decisions [16,18]. Norman Daniels and James Sabin have developed a fair process model for priority setting called 'accountability for reasonableness' (A4R) [16]. Based on justice theories of democratic deliberation, A4R identifies four conditions of a fair priority setting process (Table 1). We, and others, have been exploring the application of A4R in various health care settings [19-23]. Our experience suggests that A4R can provide valuable practical guidance to improve the fairness of actual priority setting in health care organizations and to enhance public accountability for priority setting [9,23].

To assist decision-makers in developing fair priority setting processes, we conducted one-day workshops for Board members and senior administrators at three Canadian academic health science centres (Saskatoon Health Region, Kingston General Hospital and The Ottawa Hospital), who were seeking ethics advice on how to improve priority setting in their organisations. Each organization was faced with setting priorities among their clinical services to guide resource allocation under significant budget constraints. The goal of each workshop was to help decision-makers develop a strategy for fair priority setting based on the conditions of A4R. Using case-based plenary sessions to introduce the key concepts (e.g., a case about how one organisation developed and used criteria to set clinical service priorities illustrated the importance of priority setting criteria for operationalising the Relevance condition of A4R) and facilitating consensus through small and large group discussions, we assisted workshop participants in reaching agreement on: a) the criteria decision-makers would use to set clinical service priorities, b) the processes they would follow, and c) the parameters according to which they would evaluate the success of the priority setting exercise.

We summarize key lessons learned from these workshops to help decision-makers in other health care organizations develop their own fair priority setting strategies and to improve understanding of researchers and policy makers about priority setting from the point of view of decision-makers.

## Discussion

### Presentation of lessons learned

#### Priority setting criteria

When decision-makers were asked what criteria they would use to set clinical service priorities, we found that responses clustered around eight (8) criteria (Table 2). As a step toward operationalising the Relevance condition of A4R, these criteria describe what decision-makers considered to be the most relevant decision factors (or 'reasons') for setting clinical service priorities in their organisations.

'Strategic fit' described the extent to which clinical services contributed to advancing the strategic directions of the organisation, i.e., "fit" with the organization's vision, mission, values, and goals. This criterion was consistent with the idea that strategy should be a key driver of operational planning as a counterpoint to planning based on historical or short-term political considerations.

'Alignment with external directives' identified existing government mandates and legislated obligations as relevant considerations for setting priorities. For example, each organisation had government directives to provide particular health services at prescribed volumes. This criterion recognised explicitly the limited degrees of freedom within which priorities could be set, but also highlighted the importance for decision-makers of participating with government in achieving regional and provincial health service objectives.

'Academic commitments' consisted of two sub-criteria reflective of each organization's close affiliation with a local university and medical school. The 'education' sub-criterion emphasised the role of clinical programs in educating future health care professionals and in facilitating the integration of these activities with health service delivery. The 'research' sub-criterion emphasised the role of academic health science centres in establishing best practice standards, in generating new medical knowledge (including practice-based and bench research), and in developing technological innovation. Workshop participants felt that this criterion affirmed the unique role of academic health science centres in advancing society's health care knowledge and capacity.

'Clinical impact' was defined primarily in terms of the service volumes necessary to ensure the clinical competence of medical staff to provide safe and effective care to patients. Other relevant factors included: evidence of effectiveness in health promotion and disease prevention, uniqueness of the health service in the local area, and quality of the service provided. Workshop participants expressed concern about their ability to measure clinical impact given the limitations of their institutional decision support capabilities (e.g., data, trained decision support staff). However, they felt that by identifying these factors, this could provide direction for the collection of appropriate data and information.

'Community need' described the health service needs of patients in the organisation's local catchment area. This included current demand for health services, which could be measured on the basis of utilisation rates and waiting list data, as well as future demand based on population data and trends (e.g., aging population). Community need was further defined in terms of the availability of other health service providers. For example, community need was seen to be greater if the organisation were the sole provider of a health service to patients in the region than if there were other local providers whom patients might access for care.

'Partnerships' highlighted existing formal agreements and commitments with other organisations in coordinating delivery of health care to defined populations (e.g., referral agreements to ensure access to speciality care, or transfer agreements to coordinate the transition of patients from a hospital to a chronic continuing care facility). Partnerships were seen as effective ways to enhance service quality and to optimise resource utilisation within the region or local catchment area.

'Interdependency' described the coordination and collaboration between clinical services within the organization to enhance service quality (e.g., through interdisciplinary models of care) or to use institutional resources more efficiently. In the two organisations that had achieving a "healthy" workplace as a strategic goal, workshop participants also related this criterion to quality of work life factors as key enablers of effective clinical coordination and collaboration.

'Resource implications' included a cluster of factors related to the mobilisation and use of human and fiscal resources. Although recognising that strategic planning should not be over-determined by operational issues, workshop participants felt that the resource context was relevant for setting clinical service priorities. For example, the implications of prioritisation depended in part on the source of funding (e.g., base hospital budget, ministry of health volume-based funding, donation), the availability of staff (e.g., nurses) and capital resources (e.g., equipment, space), the flexibility of contractual agreements (e.g., union contracts), and the model of health service delivery, which could be more or less efficient in using available resources.

#### Priority setting processes

When asked what key process elements would be needed in order for priority setting to be accountable and fair, workshop participants identified ten (10) elements (Table 3). Some of these process elements reflected the Publicity, Revision, and Enforcement conditions of A4R. However, decision-makers identified additional process considerations that they felt were also essential for a successful priority setting process.

Workshop participants identified a number of preparatory steps that should be taken before priority setting can begin:

(1) The organisation should establish, refine, or confirm its strategic plan. This is to ensure that the clinical service priorities that emerge through the priority setting process align with and advance the organisation's mission and strategic goals. In effect, workshop participants felt that they needed to know first where the organisation was going so that they could set the right priorities for getting there.

(2) The programmatic architecture of the organization (i.e., what services are offered and how they are grouped administratively and programmatically) should be clarified in order to set clinical service priorities relative to current activities. This step was also felt to be important for defining precisely what order of clinical service activity was to be prioritised and for creating an accurate inventory of clinical services for prioritisation.

(3) The specific responsibilities of the Board and senior management in relation to the priority setting process should be clarified explicitly and upfront. Decision-makers identified some confusion about these responsibilities given that clinical service priority setting involved an overlap of the strategic responsibility of the Board with the operational responsibility of Senior Management. During the workshop, Board members and Senior Managers drafted a memorandum of agreement delineating their respective roles and responsibilities in the priority setting process.

Workshop participants also identified a number of elements that were critical to the design of the priority setting process itself:

(4) The executive decision-making group should be multidisciplinary and its role should be clearly and explicitly defined in advance of priority setting. Workshop participants emphasised the importance of shared accountability for priority setting across the clinical and administrative leadership. Engaging the medical leadership in a decision-making role was identified as key to developing a successful priority setting process. The engagement of other non-medical clinical leaders (e.g., nursing leadership) was also thought to be important for ensuring the legitimacy of the priority setting process.

(5) Stakeholders should be engaged in the priority setting process. Although the organisational executive would ultimately be accountable for making the priority setting decisions, workshop participants felt that stakeholders could be engaged particularly as key informants through expert and broader stakeholder consultation. This consultation should include both internal stakeholders (e.g., staff, patient advisory groups) and external stakeholders (e.g., institutional partners, community groups, government officials).

(6) Priority setting criteria should be clearly defined and understood by decision-makers and stakeholders. Data/information should be collected to support their application in the priority setting process. Workshop participants felt that the criteria identified in the workshop could be further refined through stakeholder engagement and tested with decision-makers to ensure a common interpretation of each criterion and consistency in their implementation.

(7) An effective communication strategy should be developed to ensure a transparent priority setting process. The purpose of the communication strategy should be to ensure that stakeholders know and understand the scope and necessity of priority setting decision-making, the degrees of freedom within which priority setting would take place (including explicit identification of any "sacred cows" that would be immune from priority setting), and the particularities of the priority setting process (who will do what, how the process will work, and why). In addition, the rationales for priority setting decisions should be communicated to stakeholders and should clearly demonstrate how these decisions are defensible in light of the priority setting criteria and available data/information.

(8) Decision review processes should be developed to incorporate opportunities to revisit and review decisions. Workshop participants saw these as additional opportunities to engage stakeholders around difficult priority setting decisions, although they also expressed concern that this might invite conflict between stakeholders and decision-makers. However, it was generally felt that this could be mitigated if decision review processes were focused explicitly on providing a vehicle for new data/information to be brought forward, material errors in the original decision to be corrected based on available data/information, and procedural inconsistencies to be addressed.

Workshop participants identified additional elements that were important to improve quality and strengthen capacity for fair priority setting in their organisations over time:

(9) Process monitoring and formal evaluation strategies should be developed to ensure quality improvement and to realise a commitment to organizational learning. Workshop participants felt that the process should be monitored for adherence to the conditions of A4R, thus allowing for mid-course corrections to enhance fairness as the priority setting process unfolded. A formal evaluation process after priority setting would allow institutional good practices as well as opportunities for improvement to be captured so that this information could lead to improved priority setting in the future. For example, Martin & Singer have developed an ethics-based quality improvement model that focuses on evaluating and improving the fairness of priority setting processes [23].

(10) The priority setting process should be supported by leadership development and change management strategies to strengthen institutional capacity for priority setting decision-making. Capacity strengthening should focus in particular on middle managers, who may not be among the decision-making group but who would play key roles in communicating with staff and in implementing the priority setting decisions.

#### Parameters of successful priority setting

When asked how they would know that the priority setting process had been a success, workshop participants identified both outcome and process parameters (Table 4). In either case, key marks of its success were whether the process were perceived to be an improvement over past priority setting initiatives and whether it were implemented in subsequent iterations of priority setting.

Outcome parameters focused on the effects of priority setting on organizational priorities and budget, on staff, and on the community. Effects on organizational priorities and budget concerned the extent to which the priority setting process was successful in changing organizational priorities and shifting resources, in supporting and/or enhancing the mission of the organization, in contributing to conditions for growth, and in balancing the organizational budget. Effects on staff involved an evaluation of the impact of priority setting on staff satisfaction and morale, organizational recruitment and retention initiatives, and overall understanding of new priorities across the organization. Effects on the community focused on how external stakeholders, including members of the public, regional partners, health care peers (e.g., other academic health science organisations), and affiliated academic institutions, responded to the priority setting initiative.

Process parameters focused on the efficiency and fairness of the priority setting process. Efficiency of the priority setting process could be evaluated in terms of whether priority setting improved institutional capacity for allocating resources and making priority setting decisions, and whether stakeholders and decision-makers felt that the priority setting process provided a worthwhile return on the time invested to set priorities. Fairness of the priority setting process could be evaluated in terms of whether stakeholders understood and felt engaged in the priority setting process, whether priority setting decisions were justified and seen to be reasonable, and whether 'winners' and 'losers' both felt that they had been fairly treated.

It was interesting to us that, although A4R was presented as an ethical framework for fair priority setting, workshop participants did not specifically identify conformity with its conditions as a parameter of success related to fairness. The importance of these conditions is clearly evident, however, among the fairness considerations they cited as well as the process elements they identified as key to setting priorities. Moreover, we had been invited to work with these executive teams precisely because they were seeking an ethical framework through which to improve how they set priorities in their organisations. This suggests to us that A4R was seen by workshop participants primarily as an ethical framework for process design rather than for the evaluation of priority setting processes *ex post facto*.

### Implications of lessons learned

Our findings from these three priority setting workshops illuminate the complex challenges faced by decision-makers in managing scarce health care resources. The range of criteria identified in the workshops provides insight into the competing goals (e.g., clinical vs. academic, local vs. systemic, strategic vs. operational) and multiple stakeholder relationships that decision-makers must consider in setting clinical service priorities. This is consistent with previous findings that efficiency considerations or simple technical solutions have only limited influence on decision-making and are not sufficient alone to guide priority setting decision-making [8,17,24,25]. Given the range of interested stakeholders and competing values, our findings underscore the importance of procedural fairness to secure socially acceptable priority setting decisions and to ensure public accountability [8,18,26]. This suggests that a fair process model like A4R may be particularly suitable to help decision-makers set legitimate and fair clinical service priorities.

Although we report only on three health care organizations, the organisations were all academic health science centres facing similar resource challenges. Consensus around priority setting criteria and processes emerged independently among workshop participants in their large and small group discussions. However, this does not mean that these findings are exhaustive of the priority setting criteria that might be relevant for setting clinical service priorities (e.g., in community hospitals without academic affiliations) or the process elements that would be necessary to ensure a legitimate and fair priority setting process. Moreover, although our approach was based on the notion that fair priority setting requires a normative grounding in procedural justice – in this case, A4R – this does not mean that these findings are normatively 'right' for clinical service priority setting in all health care organisations. An evaluation of the normative 'rightness' depends to some extent on the specific institutional circumstances under which priority setting is taking place, the stakeholders who are affected, and the strategic goals that are being pursued. Experience shows, moreover, that the conditions of A4R are sufficiently general to guide fair priority setting in various institutional settings [9,16,20,27]. Thus, decision-makers in other health care organisations may draw lessons from these workshops to operationalise fair priority setting processes that reflect the particularities of their institutional circumstances and ensure accountability for the reasonableness of their clinical service priorities.

Our experience shows that, from the perspective of Board members and senior leaders, our practical approach using A4R offers useful guidance for developing fair and publicly accountable priority setting processes under resource constraints. However, alternative priority setting approaches may also be beneficial. For example, program budgeting and marginal analysis, an economics-based approach, has been used with senior health care administrators in Canada and elsewhere to improve how priority setting optimises health and non-health benefits within available resources [13]. A comparison of priority setting approaches has not been done, however preliminary work has begun to explore a more interdisciplinary priority setting approach (Gibson JL, Mitton C, Martin DK, Donaldson C, Singer PA, manuscript submitted) [21].

Despite these possible limitations, the lessons we report here fill an important gap in the literature about the criteria, processes, and parameters of success decision-makers would use to set priorities using an ethical framework. We expect that decision-makers in other health care organizations may find themselves in the workshop participants' experience of priority setting and may use these findings as a basis for discussing how they could enhance the fairness and public accountability of their own priority setting processes.

## Summary

• Hospitals and regional health authorities must set priorities in the face of resource constraints.

• Decision-makers seek pragmatic ways to set priorities fairly in strategic planning, but find limited guidance from the literature.

• We facilitated workshops for board members and senior leadership at three organizations to assist them in developing a strategy for fair priority setting.

• Workshop participants identified 8 priority setting criteria, 10 key priority setting process elements, and 6 parameters of success that they would use to set priorities in their organizations.

• Decision-makers in other organizations can draw lessons from these findings to enhance the fairness of their priority setting decision-making.

## Competing interests

The authors were compensated by the health care organizations for facilitating the priority setting workshops and continue to consult with these and other health care organizations.

## Authors' contributions

JLG conducted the workshops on which this paper is based, collated and analysed the data, and drafted the manuscript.

DKM participated in analysing the data and commented on earlier drafts of the manuscript.

PAS conducted the workshops on which this paper is based, participated in analysing the data, commented on earlier drafts of the manuscript, and conceived of the paper.

All authors read and approved the final manuscript.

## Pre-publication history

The pre-publication history for this paper can be accessed here:



## Figures and Tables

**Table 1:** Accountability for reasonableness^16^

Relevance condition	Decisions should be made on the basis of reasons (i.e., evidence, principles, arguments) that "fair-minded" people can agree are relevant under the circumstances.
Publicity condition	Decisions and their rationales should be transparent and made publicly accessible.
Revision condition	There should be opportunities to revisit and revise decisions in light of further evidence or arguments, and there should be a mechanism for challenge and dispute resolution.
Enforcement condition	There should be either voluntary or public regulation of the process to ensure that the other three conditions are met.

**Table 2:** Priority setting criteria

• Strategic fit
• Alignment with external directives
• Academic commitments
– Education
– Research
• Clinical impact
• Community needs
• Partnerships (external)
• Interdependency (internal)
• Resource implications

**Table 3:** Priority setting process elements

• Confirm the strategic plan
• Clarify programmatic architecture, including program groupings and definitions
• Clarify Board/Mgmt roles and responsibilities
• Determine who will make priority setting decisions and what they will do
• Engage internal/external stakeholders
• Define priority setting criteria and collect data/information
• Develop an effective communication strategy
• Develop a decision review process
• Develop process monitoring and evaluation strategies
• Support the process with leadership development and change management strategies

**Table 4:** Parameters of success

Outcome parameters	Process parameters
*Effect on organizational priorities and budget*	*Efficiency of priority setting process*
• Priorities change; resource shift	• Increased ease in allocating resources
• Strategic plan supported/enhanced	• Improved capacity for making priority setting decisions
• Conditions for growth created/enhanced	• Perceived return on time invested
• Budget balanced	

*Effect on staff*	*Fairness*
• Staff satisfaction neutral or positive	• Stakeholders understand the process
• Staff retention/recruitment neutral or positive	• Stakeholders feel engaged
• Organizational understanding improved	• Priorities are justified and seen to be reasonable
	• Process is perceived to be consistent and fair
	• Winners/losers issue well-managed

*Effect on community*	*Conformity with conditions of 'accountability for reasonableness'?*
• Public media recognition neutral or positive	
• Public acceptance or community support improved	
• Public perception of institutional accountability improved	
• Health care integration through partnerships increased	
• Education/research peer recognition enhanced	
• Emulated by other organizations	
